# Supplementary material for: Identifying metabolic reprogramming phenotypes with glycolysis-lipid metabolism discoordination and intercellular communication for lung adenocarcinoma metastasis
Source: Commun Biol. 2022 Mar 17;5:198. doi: 10.1038/s42003-022-03135-z (PMC8931047; doi:10.1038/s42003-022-03135-z)
Supplement: Supplementary file 2 — Supplementary Information [file 42003_2022_3135_MOESM2_ESM.pdf]

Supplementary information for

Identifying metabolic reprogramming phenotypes with glycolysis and lipid metabolism discoordination and intercellular communication for lung adenocarcinoma metastasis

Running title: Metabolic reprogramming phenotypes for lung adenocarcinoma metastasis

This PDF file includes:

Supplementary Tables 1-5

Supplementary Figures 1-6

Supplementary Tables

Supplementary Table 1. The detailed functions of the 11 DE genes

Gene id	Gene symbol	Pathway	Description	FC	*P* value	FDR
226	*ALDOA*	Glycolysis / Gluconeogenesis	Aldolase A (ALDOA) is a glycolytic enzyme that catalyzes the reversible conversion of fructose-1, 6-bisphosphate to glyceraldehyde 3-phosphate and dihydroxyacetone phosphate.	1.03	3.15E-04	0.0295
2023	*ENO1*	Glycolysis / Gluconeogenesis	Alpha-enolase (ENO1), one of the three major enolases, is a key regulatory enzyme in glycolysis. And ENO1 is also involved in transcription regulation, cell differentiation process and catalyze glycolysis.	1.03	2.37E-04	0.0276
2597	*GAPDH*	Glycolysis / Gluconeogenesis	Glyceraldehyde-3-phosphate dehydrogenase (GAPDH), is a key enzyme involved in glycolysis that participates in the conversion of glyceraldehyde-3-phosphate (G3P) to 1,3-bisphosphoglycerate in the cytoplasm, thereby regulating the production of ATP for energy metabolism.	1.04	1.03E-05	0.0116
2821	*GPI*	Glycolysis / Gluconeogenesis	Glucose-6-phosphate isomerase (GPI) is a second glycolytic enzyme that catalyzes the isomerisation of G6P into fructose-6-phosphate.	1.04	2.04E-04	0.0265
3939	*LDHA*	Glycolysis / Gluconeogenesis	Lactate dehydrogenase A (LDHA), is a key enzyme that converts pyruvate into lactic acid during glycolysis, this gene catalyzes the conversion of L-lactate and NAD to pyruvate and NADH in the final step of anaerobic glycolysis.	1.04	3.58E-05	0.0153
5223	*PGAM1*	Glycolysis / Gluconeogenesis	Phosphoglycerate mutase 1 (PGAM1), as one of the important enzymes in the glycolysis pathway, catalyzes the conversion of 3-phosphoglycerate (3-PG) in the glycolysis pathway to 2-phosphoglycerate (2-PG), promote glucose metabolism and energy production.	1.06	4.15E-05	0.0163
55276	*PGM2*	Glycolysis / Gluconeogenesis	Phosphoglucomutase 2	1.09	1.66E-04	0.0244

7167	*TPI1*	Glycolysis / Gluconeogenesis	Triosephosphate isomerase (TPI) is the most active enzyme in the glycolytic pathway, catalyzes the isomerization of glyceraldehydes 3-phosphate (G3P) and dihydroxy-acetone phosphate (DHAP) in glycolysis and gluconeogenesis.	1.03	3.91E-04	0.0326
8611	*PLPP1*	Glycerophospholipid metabolism, Ether lipid metabolism	Phospholipid phosphatase 1 (PLPP1) is a member of the phosphatidic acid phosphatase (PAP) family. PAP converts phosphatidic acid to diacylglycerol and plays a role in glycerolipid synthesis and phospholipase D-mediated signal transduction.	0.94	8.98E-04	0.0450
23171	*GPD1L*	Glycerophospholipid metabolism	Glycerol-3-phosphate dehydrogenase 1 like (GPD1L) catalyzes the conversion of sn-glycerol 3-phosphate to glycerone phosphate	0.92	4.19E-04	0.0334
23646	*PLD3*	Glycerophospholipid metabolism, Ether lipid metabolism	PLD3 (phospholipase D family, member 3) gene encodes a lysosomal protein that belongs to the phospholipase D (PLD) superfamily, which catalyzes the hydrolysis of membrane phospholipids.	0.95	1.56E-04	0.0238

Supplementary Table 2. Clinical and molecular characteristic differences between metabolic reprogramming phenotypes (MPs)

	Characteristics	MP-I	MP-II	MP-III	*P* value
**Stage** (number, percentage)	I	107 (58.15%)	50 (37.59%)	19 (26.76%)	8.49E-06
	II	39 (21.2%)	45 (33.83%)	19 (26.76%)	
	III	31 (16.85%)	31 (23.31%)	22 (30.99%)	
	IV	7 (3.80%)	7 (5.26%)	11 (15.49%)	
**Age** (number, percentage)	< 65	80 (42.78%)	63 (46.32%)	39 (54.93%)	0.22
	≥ 65	107 (57.22%)	73 (53.68%)	32 (45.07%)	
**Gender** (number, percentage)	Female	101 (54.01%)	70 (51.47%)	34 (47.89%)	0.66
	Male	86 (45.99%)	66 (48.53%)	37 (52.11%)	
**Transcriptomics Subtypes** (number, percentage)	Bronchioid	124 (66.31%)	37 (27.21%)	2 (2.82%)	1.87E-26
	Squamoid	21 (11.23%)	38 (27.94%)	12 (16.9%)	
	Magnoid	42 (22.46%)	61 (44.85%)	57 (80.28%)	
**Molecular scores** (z-score median)	Hypoxia score	-0.6773	0.3982	1.0821	2.77E-37
	Stemness score	-0.5642	0.2347	0.7599	2.18E-31
	Proliferation score	-0.6003	0.2439	0.6067	4.07E-30
	Immune score	0.0743	0.2419	-0.3013	1.77E-02
	TMB	-0.4424	-0.3378	0.0482	7.49E-05
**Genetic lesions** (number, percentage)	*TP53* mutation	66 (35.86%)	75 (56.39%)	48 (67.61%)	2.36E-06
	KEAP1 mutation	26 (14.13%)	15 (11.28%)	28 (39.44%)	3.65E-06
	SETD2 deletion	37 (20.11%)	36 (20.07%)	30 (42.25%)	2.06E-03
	PBRM1 deletion	37 (20.11%)	36 (20.07%)	30 (42.25%)	2.06E-03

Note: MP represents metabolic reprogramming phenotype

Supplementary Table 3. Differences in genetic lesions of 28 LUAD driver genes across the MPs

Gene id	Gene symbol	Genetic lesions	Fisher test *P*	FDR
7157	*TP53*	Gene mutation	2.36E-06	7.30E-05
9817	*KEAP1*	Gene mutation	3.65E-06	7.30E-05
29072	*SETD2*	CNV deletion	0.0021	0.0206
55193	*PBRM1*	CNV deletion	0.0021	0.0206
4233	*MET*	CNV amplification	0.0065	0.0519
3845	*KRAS*	CNV amplification	0.0121	0.0723
2058	*EPRS*	Gene mutation	0.0127	0.0723
6794	*STK11*	Gene mutation	0.0165	0.0826
5290	*PIK3CA*	Gene mutation	0.0241	0.1070
1956	*EGFR*	CNV amplification	0.0321	0.1284
4089	*SMAD4*	CNV deletion	0.0363	0.1320
1499	*CTNNB1*	Gene mutation	0.0808	0.2659
5728	*PTEN*	Gene mutation	0.0864	0.2659
4233	*MET*	Gene mutation	0.0986	0.2816
9612	*NCOR2*	CNV deletion	0.1278	0.3409
55729	*ATF7IP*	CNV deletion	0.1517	0.3792
5925	*RB1*	Gene mutation	0.1706	0.3792
29072	*SETD2*	Gene mutation	0.1615	0.3792
23269	*MGA*	CNV deletion	0.2203	0.4197
9476	*NAPSA*	CNV deletion	0.2152	0.4197
9612	*NCOR2*	Gene mutation	0.2115	0.4197
5921	*RASA1*	Gene mutation	0.2497	0.4541
8241	*RBM10*	Gene mutation	0.2635	0.4546
1130	*LYST*	Gene mutation	0.2799	0.4546
4089	*SMAD4*	Gene mutation	0.2841	0.4546
85415	*RHPN2*	Gene mutation	0.2969	0.4568
1956	*EGFR*	Gene mutation	0.3371	0.4919
3845	*KRAS*	Gene mutation	0.3443	0.4919
143	*PARP4*	Gene mutation	0.4145	0.5527
673	*BRAF*	Gene mutation	0.4121	0.5527
324	*APC*	Gene mutation	0.4392	0.5667
23269	*MGA*	Gene mutation	0.5140	0.6426
55729	*ATF7IP*	Gene mutation	0.5369	0.6507
55193	*PBRM1*	Gene mutation	0.5785	0.6806
4763	*NF1*	Gene mutation	0.6954	0.7947
7750	*ZMYM2*	Gene mutation	0.8603	0.9559
324	*APC*	CNV deletion	0.9350	0.9842
5921	*RASA1*	CNV deletion	0.9350	0.9842
9476	*NAPSA*	Gene mutation	1.0000	1.0000

7270	*TTF1*	Gene mutation	1.0000	1.0000

Supplementary Table 4. Nine ligands significantly differently expressed in MP-III compared to MP-I in the two scRNA-seq datasets

Gene	GSE131907			GSE123902		
	FC	*P*	FDR	FC	*P*	FDR
ANGPTL4	2.95	2.03E-24	5.47E-23	1.97	2.91E-05	1.16E-04
EREG	1.69	3.33E-16	8.67E-15	INF	2.95E-03	5.90E-03
MIF	1.43	1.10E-36	3.20E-35	1.46	2.08E-07	2.08E-06
VEGFA	1.40	7.47E-08	1.72E-06	1.79	7.45E-11	1.49E-09
ANXA1	1.39	2.09E-25	5.86E-24	1.43	1.77E-05	1.06E-04
MDK	1.26	1.25E-14	3.12E-13	2.15	1.71E-16	4.97E-15
AREG	1.21	2.94E-06	6.18E-05	2.24	2.32E-16	6.49E-15
LGALS9	1.19	2.70E-04	4.85E-03	3.94	2.07E-12	5.16E-11
HBEGF	1.13	2.44E-03	3.66E-02	2.28	2.37E-11	5.68E-10

Supplementary Table 5. Baseline clinical characteristics of stage I patients treated with curative surgery resection only

Data Source	Year	Sample	Age		Gender	
			Age < 65	Age ≥ 65	Female	Male
TCGA	2014	122	45	77	66	56
GSE31210	2011	162	114	48	93	69
GSE50081	2013	92	28	64	46	46
GSE13213	2009	79	50	29	38	41
GSE42127	2013	67	32	35	38	29
GSE68465	2015	223	102	121	114	109

Supplementary Figures

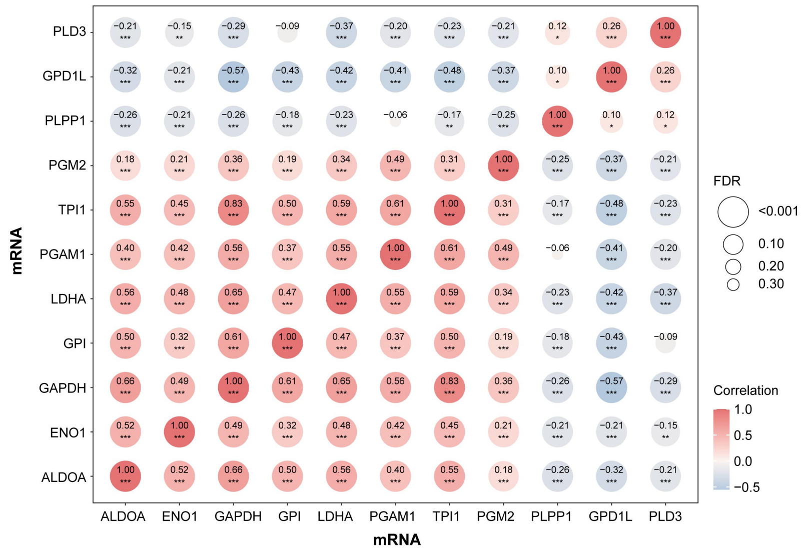

Supplementary Figure 1. Correlation between the mRNA expression of the 11 metabolic genes.

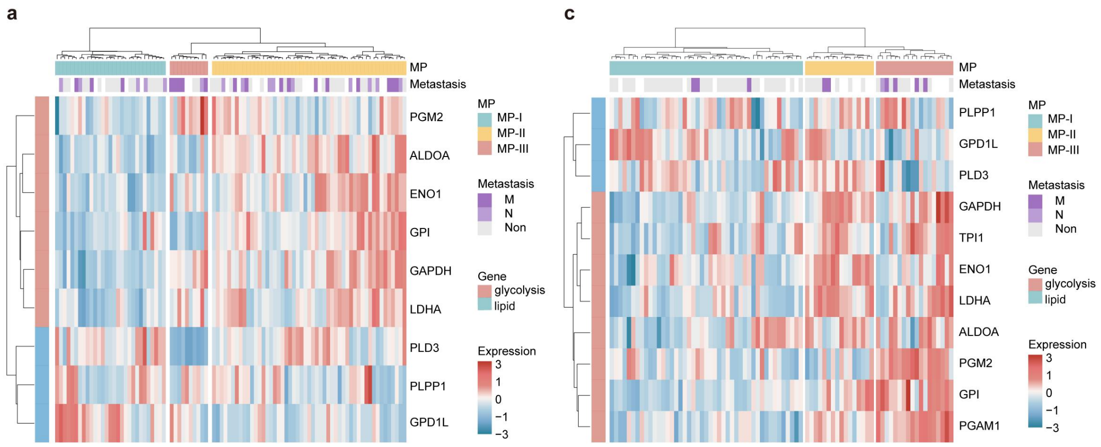

b

	MP- I	MP- II	MP- III
**M**	**3(13.04%)**	10(27.03%)	**5(62.50%)**
**N**	5(21.74%)	9(24.32%)	1(12.50%)
**Non**	**15(65.22%)**	18(48.65%)	**2(25%)**

Fisher *P* = 0.0169

d

	MP- I	MP- II	MP- III
**M**	**3(8.11%)**	2(22.22%)	**4(30.77%)**
**N**	0(0%)	0(0%)	1(7.69%)
**Non**	**34(91.89%)**	7(77.78%)	**8(61.54%)**

Fisher *P* = 0.0513

Supplementary Figure 2. Metabolic reprogramming phenotypes (MPs) in the GSE11969 and GSE13213 dataset. (**a**) Hierarchical clustering heatmap based on the mRNA expression (Z-score) of the 11 metabolic genes in the GSE11969 dataset. **(b)** Confusion Matrix for the numbers of distant metastatic (M), lymph node metastasis (N), and non-metastatic (Non) samples in the different MPs in the GSE11969 dataset. (**c**) Hierarchical clustering heatmap based on the mRNA expression (Z-score) of the

11 metabolic genes in the GSE13213 dataset. **(d)** Confusion Matrix for the numbers of distant metastatic (M), lymph node metastasis (N), and non-metastatic (Non) samples in the different MPs in the GSE13213 dataset.

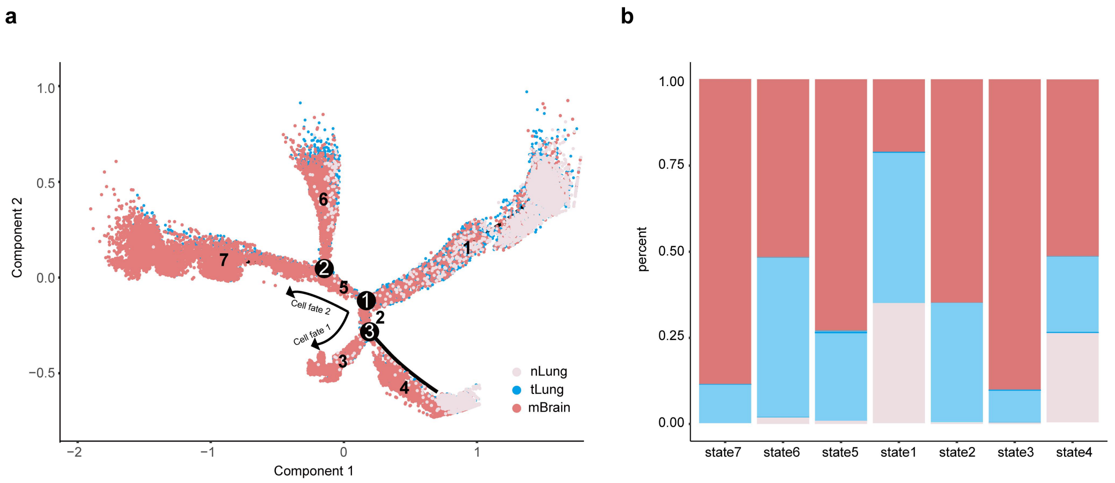

Supplementary Figure 3. Evolutionary trajectory of epithelial cells from normal to mBrain tissues. (**a**) Unsupervised transcriptional trajectory of epithelial cells based on the 11 metabolic genes using Monocle, coloured based on tissues. **(b)** The percentage of epithelial cells from each tissue in State 1-State 7.

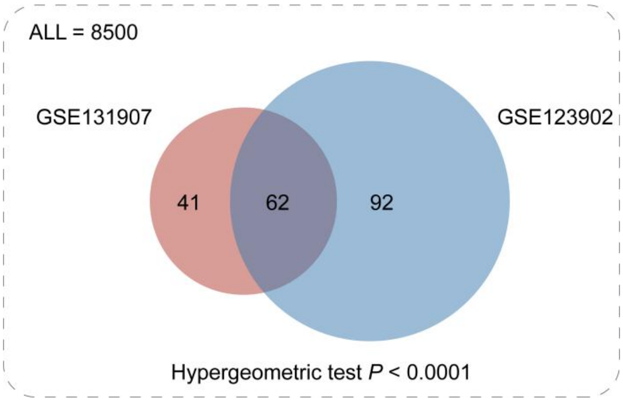

Supplementary Figure 4. Validation of cell-cell communications in the GSE123902 dataset. Venn diagram displays the number of ligand-receptor interactions that MP-III interacting with other cells in the GSE131907 dataset (red circle) and the GSE123902 dataset (blue circle). “ALL” represents the number of all possible interactions.

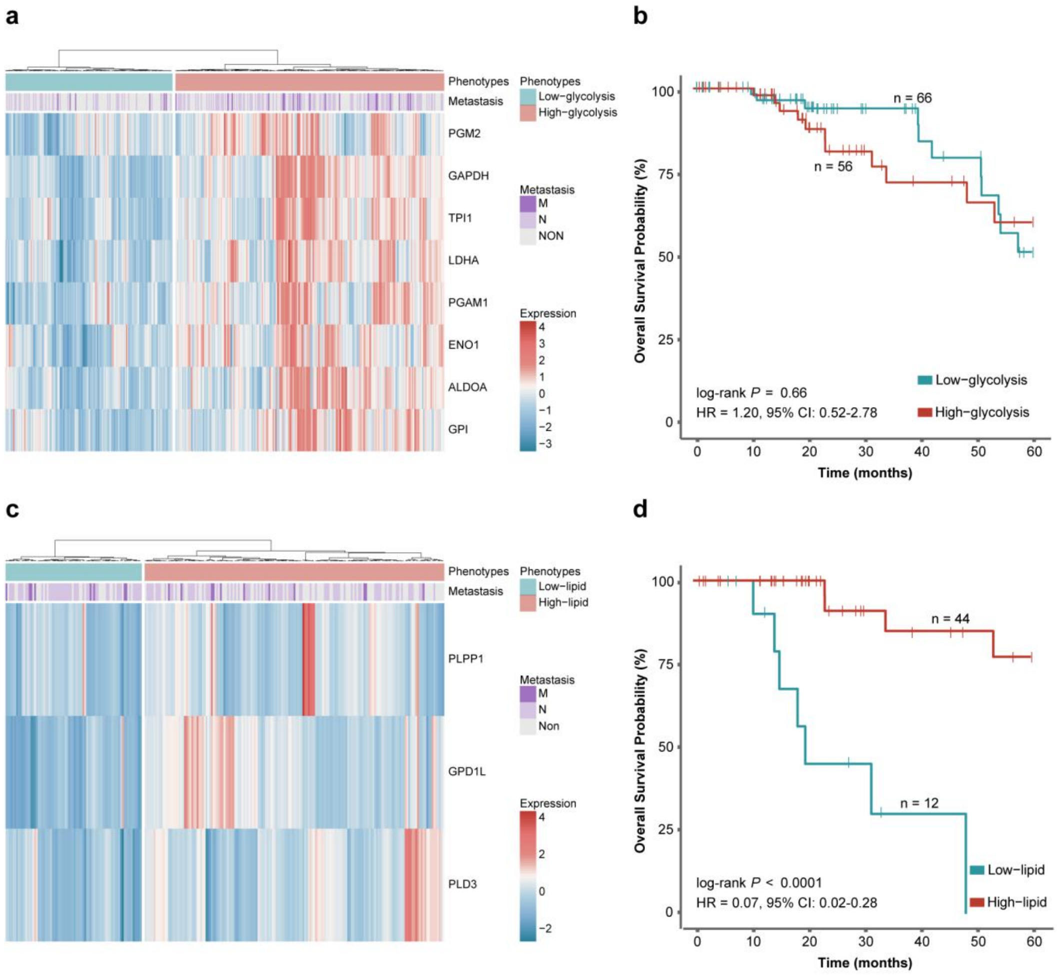

Supplementary Figure 5. Confirming of phenotypes only based on glycolytic or lipid metabolic genes in the TCGA dataset. (a) Hierarchical clustering heatmap for the 394 samples based on the mRNA expression (Z-score) of the eight glycolytic genes. **(b)** Kaplan–Meier curves of overall survival for 122 samples obtained from treatment-naive patients with stage I LUAD. **(c)** Hierarchical clustering heatmap for the 243 high-glycolysis samples based on the mRNA expression (Z-score) of the three lipid metabolic genes. **(d)** Kaplan–Meier curves of overall survival for 56 samples obtained from treatment-naive patients with stage I LUAD.

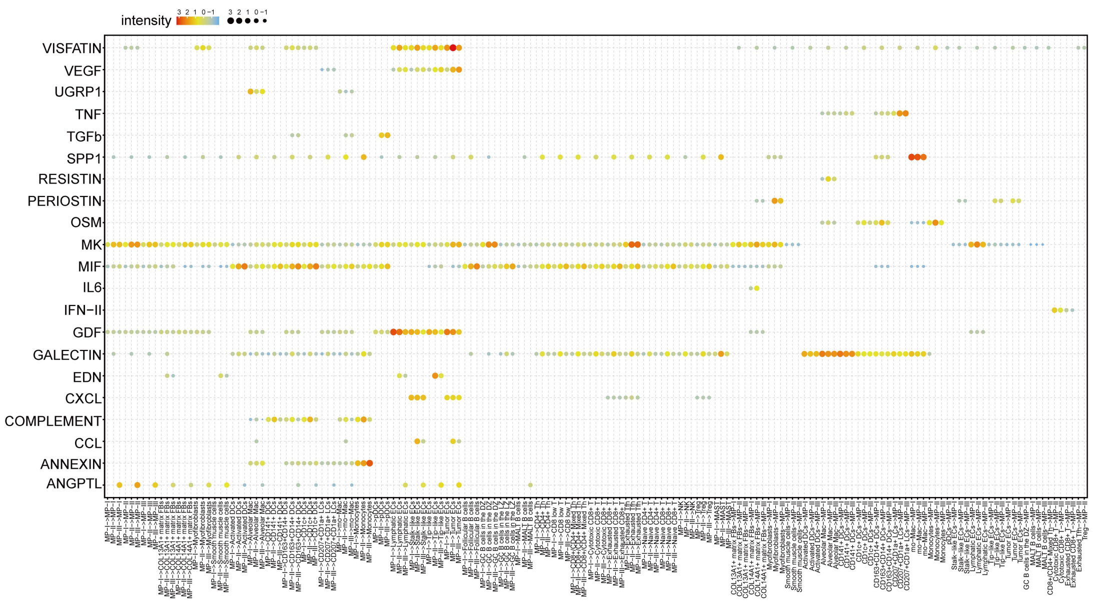

Supplementary Figure 6. Crosstalk intensity heatmap for corresponding signaling pathways of ligand-receptor pairs in subdivided cell subtypes. The subdivided cell subtypes include Fibroblasts (COL13A1$^+$ matrix FBs, COL14A1$^+$ matrix FBs, Myofibroblasts, Smooth muscle cells), Myeloid cells (Activated DCs, Alveolar Mac, CD141$^+$ DCs, CD163$^+$CD14$^+$ DCs, CD1c$^+$ DCs, CD207$^+$CD1a$^+$ LCs, mo-Mac, Monocytes, pDCs), Endothelial cells (Stalk-like ECs, Lymphatic ECs, Tip-like ECs, Tumor ECs), B lymphocytes (Follicular B cells, GC B cells in the DZ, GC B cells in the LZ, MALT B cells), T lymphocytes (CD4$^+$ Th, CD8 low T, CD8$^+$/CD4$^+$ Mixed Th, Cytotoxic CD8$^+$ T, Exhausted CD8$^+$ T, Exhausted Tfh, Naive CD4$^+$ T, Naive CD8$^+$ T, Treg), NK, and MAST cells. The sophisticated interactome of the MPs with refined subdivided cell subtypes is shown in Supplementary Date 3.
