# Supplementary material for: Identifying metabolic reprogramming phenotypes with glycolysis-lipid metabolism discoordination and intercellular communication for lung adenocarcinoma metastasis
Source: Commun Biol. 2022 Mar 17;5:198. doi: 10.1038/s42003-022-03135-z (PMC8931047; doi:10.1038/s42003-022-03135-z)
Supplement: Supplementary file 3 — Description of Additional Supplementary Files [file 42003_2022_3135_MOESM3_ESM.pdf]

Description of Additional Supplementary Files

File name: Supplementary Data 1
Description: One hundred and three ligand-receptor pairs interacting MP-III with other cell populations in the GSE131907 dataset.

File name: Supplementary Data 2
Description: Pearson correlation between glycolytic and lipid metabolic genes with MPs in the TCGA dataset.

File name: Supplementary Data 3
Description: Ligand-receptor pairs interacting MP-III with refined subdivided cell subtypes in the GSE131907 dataset.

File name: Supplementary Data 4
Description: Source data underlying some important graphs.
